# GLP-1 Analog Liraglutide Improves Vascular Function in Polymicrobial Sepsis by Reduction of Oxidative Stress and Inflammation

**DOI:** 10.3390/antiox10081175

**Published:** 2021-07-23

**Authors:** Johanna Helmstädter, Karin Keppeler, Franziska Aust, Leonie Küster, Katie Frenis, Konstantina Filippou, Ksenija Vujacic-Mirski, Simeon Tsohataridis, Sanela Kalinovic, Swenja Kröller-Schön, Matthias Oelze, Markus Bosmann, Thomas Münzel, Andreas Daiber, Sebastian Steven

**Affiliations:** 1 Center for Cardiology, Department of Cardiology 1–Molecular Cardiology, University Medical Center, 55131 Mainz, Germany; johanna.helmstaedter@uni-mainz.de (J.H.); karin.keppeler@uni-mainz.de (K.K.); fpawelke@students.uni-mainz.de (F.A.); kuester.leonie@gmail.com (L.K.); katiefrenis@gmail.com (K.F.); kfilippo@students.uni-mainz.de (K.F.); ksenija.vujacic.mirski@gmail.com (K.V.-M.); simeon.tsohataridis@gmx.de (S.T.); sanelakalinovic@gmail.com (S.K.); swenja.kroeller-schoen@gmx.de (S.K.-S.); matthias.oelze@unimedizin-mainz.de (M.O.); tmuenzel@uni-mainz.de (T.M.); daiber@uni-mainz.de (A.D.); 2 Center for Thrombosis and Hemostasis, University Medical Center of the Johannes Gutenberg-University, Langenbeckstr. 1, 55131 Mainz, Germany; markus.bosmann@unimedizin-mainz.de; 3 Pulmonary Center, Department of Medicine, Boston University School of Medicine, Boston, MA 02118, USA; 4 German Center for Cardiovascular Research (DZHK), Partner Site Rhine-Main, Langenbeckstr. 1, 55131 Mainz, Germany

**Keywords:** glucagon-like peptide-1 (GLP-1), incretins, liraglutide, peritoneal and polymicrobial sepsis, cecal ligation and puncture (CLP), endothelial dysfunction, oxidative stress, vascular inflammation

## Abstract

Sepsis causes high mortality in the setting of septic shock. LEADER and other trials revealed cardioprotective and anti-inflammatory properties of glucagon-like peptide-1 (GLP-1) analogs like liraglutide (Lira). We previously demonstrated improved survival in lipopolysaccharide (LPS)-induced endotoxemia by inhibition of GLP-1 degradation. Here we investigate the effects of Lira in the polymicrobial sepsis model of cecal ligation and puncture (CLP). C57BL/6J mice were intraperitoneally injected with Lira (200 µg/kg/d; 3 days) and sepsis induced by CLP after one day of GLP-1 analog treatment. Survival and body temperature were monitored. Aortic vascular function (isometric tension recording), protein expression (immunohistochemistry and dot blot) and gene expression (qRT-PCR) were determined. Endothelium-dependent relaxation in the aorta was impaired by CLP and correlated with markers of inflammation (e.g., interleukin 6 and inducible nitric oxide synthase) and oxidative stress (e.g., 3-nitrotyrosine) was higher in septic mice, all of which was almost completely normalized by Lira therapy. We demonstrate that the GLP-1 analog Lira ameliorates sepsis-induced endothelial dysfunction by the reduction of vascular inflammation and oxidative stress. Accordingly, the findings suggest that the antioxidant and anti-inflammatory effects of GLP-1 analogs may be a valuable tool to protect the cardiovascular system from dysbalanced inflammation in polymicrobial sepsis.

## 1. Introduction

Liraglutide is a long-acting analog of the peptide hormone glucagon-like peptide-1 (GLP-1), which is released after food uptake and is involved in the regulation of glucose hemostasis [1]. Native GLP-1 and its analogs (e.g., liraglutide, semaglutide and albiglutide) stimulate the release of insulin from beta-cells in the pancreas but since the GLP-1 receptor (GLP-1R) is not only expressed in the pancreas, they have also effects on other organs/tissues [2,3]. Clinical trials designed to test the cardiovascular safety of the new antidiabetic drug class of GLP-1 analogs surprisingly revealed that treatment of type 2 diabetes mellitus with a long-acting GLP-1 analog leads to less cardiovascular events compared to standard care [4]. The mechanism is not fully understood, but several clinical and preclinical studies indicate that GLP-1 analogs inhibit inflammatory processes [5,6]. 

Plasma levels of native GLP-1 are elevated in critically ill patients and after myocardial infarction [7,8], which might indicate that GLP-1 is also released upon inflammatory stimuli aiming at counteracting the inflammatory cascade. On the other hand, GLP-1 signaling as a potential pharmaceutical target to improve sepsis has already been preclinically tested. We and others demonstrated that GLP-1 analogs like liraglutide and inhibitors of the enzyme dipeptidylpeptidase-4 (DPP-4), which rapidly degrades native GLP-1, ameliorate cardiovascular and thrombotic complications in animal models of lipopolysaccharide (LPS)-induced sepsis [9,10,11,12]. Mechanistically, the GLP-1 analog and DPP-4 inhibition suppressed inflammation and formation of reactive oxygen species (ROS) in the vasculature, all of which led to an improved endothelium-dependent vasorelaxation and normalization of hypotension [11]. We further examined the effect of the GLP-1 analog liraglutide on microvascular thrombosis and platelet reactivity in LPS-induced sepsis and observed less organ damage by thrombotic occlusion of the microvascular circulation in the lung, which might be secondary to improved endothelial function and bioavailability of nitric oxide (^•^NO) [12]. On the other hand, we and others also found some direct inhibitory effects of liraglutide on thrombocytes [12,13], although the expression of the GLP-1R on platelets is a matter of debate within the scientific community [14]. 

Sepsis is a severe disease and associated with a high mortality. The basic treatment regimen for sepsis did not change for decades. Early fluid supplementation and antibiotics are known to significantly improve survival in septic shock, but clinical trials targeting the reduction of the inflammatory response failed. Our results from previous studies indicate that GLP-1 analogs might help to improve septic complications and mortality, however, the murine and rat models of LPS-induced sepsis have several limitations [15] and may be difficult to translate to human sepsis. 

In the present study, we investigated the effects of the GLP-1 analog liraglutide in polymicrobial sepsis induced by cecal ligation and puncture (CLP). Using this clinically relevant animal sepsis model, we observed improved endothelial function by reduction of vascular inflammation and oxidative stress under liraglutide therapy. These results indicate that anti-inflammatory actions of GLP-1 receptor activation might represent a new therapeutic concept to treat the cardiovascular complications of deleterious sepsis.

## 2. Materials and Methods

### 2.1. Chemicals

Liraglutide was obtained as an injection pen (6 mg/mL from Victoza/Novo Nordisk, Bagsværd, Denmark). Prostaglandin F2α for isometric tension studies was purchased from Cayman Chemical (Ann Arbor, MI, USA). L-012 (8-amino-5-chloro-7-phenylpyrido[3,4-d]pyridazine-1,4-(2H,3H)dione sodium salt) was used from Wako Pure Chemical Industries (Osaka, Japan) and sodium citrate solution (3,13%) from Eifelfango (Bad Neuenahr-Ahrweiler, Germany). The Bradford reagent was obtained from BioRad (Hercules, CA, USA). The QuantiTect Probe RT-PCR Kit was purchased from Qiagen (Hilden, Germany) and TaqMan probes from Applied Biosystems (Waltham, MA, USA). All other chemicals were of analytical grade and obtained from Sigma-Aldrich (St. Louis, MO, USA), Fluka/Honeywell (Charlotte, NC, USA) or Merck (Darmstadt, Germany).

### 2.2. Animals

All animals were treated in accordance with the Guide for the Care and Use of Laboratory Animals as adopted by the U.S. National Institutes of Health and approval was granted by the Ethics Committee of the University Hospital Mainz and the Landesuntersuchungsamt Rheinland-Pfalz (Koblenz, Germany; permit number: 23 177-07/G 14-1-039). All mice were male C57BL/6J between 8 and 12 weeks old, purchased from Charles River. The mice were housed in a 12-hour light/dark cycle in the institutional animal facility with free access to food and water. 

**Figure 1:**
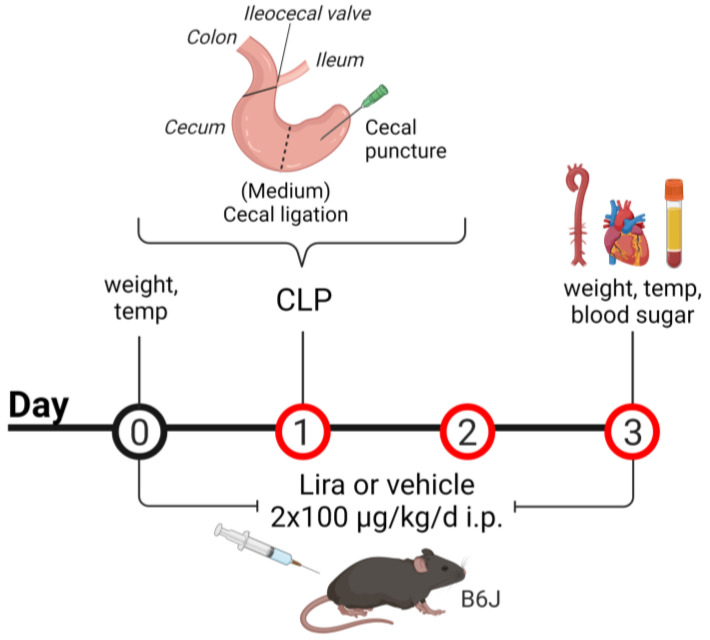
Scheme for treatment and cecal ligation and puncture (CLP) procedure. Male C57BL/6J mice were treated with liraglutide (Lira) or the vehicle (saline) twice daily, every 12 h, for 3 consecutive days via intraperitoneal injections (i.p.) at a dose of 2 × 100 µg/kg/d for Lira or an adequate volume of the vehicle. One day after initial administration, a mid-grade polymicrobial sepsis was induced by cecal ligation at the medium position below the ileocecal valve, followed by needle perforation (puncture) of the cecum. Created with BioRender.com.

Three groups of mice were used: vehicle-treated and sham-operated controls (B6J), vehicle-treated septic mice induced by cecal ligation and puncture (CLP) and liraglutide-treated mice subjected to CLP (+Lira). Intraperitoneal injections of liraglutide (100 µ/kg) or saline (vehicle, adequate volume for B6J and CLP group) were started one day prior to the CLP procedure and continued until euthanasia, with an application twice daily every 12 h. A total of 48 h following sepsis induction by CLP, animals were sacrificed by cardiac puncture after anesthesia with ketamine/xylazine (120 mg/kg ketamine and 16 mg/kg xylazine in 0.9% NaCl). The heart, aorta and blood were harvested. Glucose levels were assessed in whole blood using the ACCU-CHEK sensor system from Roche Diagnostics GmbH (Basel, Schweiz). The mice were weighed before treatment and before euthanasia and their body temperature determined using an infrared thermometer for small rodents from Bioseb (Vitrolles, France) (Figure 1).

### 2.3. CLP Procedure

The cecal ligation and puncture (CLP) method was used as an experimental model for the induction of a polymicrobial sepsis in rodents. The CLP procedure comprises the cecal ligation below the ileocecal valve, followed by needle perforation (puncture) of the cecum [16]. Since rodent sepsis induced by CLP mimics the pathogenesis (bacteremia evoking the host immune response, leading to septic shock, multiorgan failure and finally death) and symptoms of the human disease well, it is considered a realistic and therefore clinically relevant sepsis model [16,17,18]. The surgical procedure was performed according to the published protocol by Rittirsch et al. [18]. In short, the cecum of the mice was exposed after midline laparotomy under a ketamine/xylazine anesthesia (120 mg/kg ketamine and 16 mg/kg xylazine in 0.9% NaCl). The cecum was ligated at the medium position for the induction of a midgrade sepsis with the ileocecal valve as a reference to determine the cecal length (Figure 1). Using a 21-gauge needle, the cecum was punctured in a mesenteric-to-antimesenteric direction. A small droplet of feces was milked into the peritoneum to induce bacterial peritonitis. After relocating the cecum into the abdominal cavity, the wound was closed by running sutures to the abdominal musculature and to the skin. After completed surgery, animals were resuscitated by injection of prewarmed saline (37 °C, 50 mL/kg, s.c.). Sham animals underwent the same surgical procedure except for the cecal ligation and puncture step. Mice were monitored closely after surgery and buprenorphine (0.05 mg/kg, s.c.) was injected every 6 h for postoperative analgesia (until euthanasia). For best consistency and reproducibility, the procedure was done at the same time of the day and performed by the same operator.

### 2.4. Isometric Tension Recordings

For vascular reactivity studies, aortas were dissected from the mice and cleaned of perivascular fat. The 4-mm segments of thoracic aortas were cut and mounted on force transducers within the organ bath chamber. The rings were subjected to a KCl bolus of 80 mM, washed and a constriction-curve in response to increasing concentrations of depolarizing KCl was performed. To test vascular responses to the endothelium-dependent vasodilator acetylcholine (ACh) and to the endothelium-independent vasodilator nitroglycerine (NTG), vessels were preconstricted by 2 µM prostaglandin F2α to yield approximately 70–80% of the maximal tone induced by KCl bolus and concentration–relaxation curves were recorded as previously described [19,20].

### 2.5. Detection of Oxidative Stress in Whole Blood, Plasma, Cardiac and Aortic Tissue

A whole blood oxidative burst was used as an indicator of the global pro-oxidant burden and activation of inflammatory pathways because it mainly reflects leukocyte NADPH oxidase (Nox) and myeloperoxidase activity [21,22,23]. Fresh citrate-anticoagulated blood (1:10) was stimulated with phorbol dibutyrate (PDBu, 10 μM) and assessed in PBS containing Ca^2+/^Mg^2+^ (1 mM) with L-012 (100 μM)-enhanced chemiluminescence (ECL) by a Mithras LB 943 plate reader (Berthold Technologies, Bad Wildbad, Germany). A PDBu-induced oxidative burst was normalized to the number of leukocytes in the whole blood, assessed with an automated hematology analyzer (Sysmex KX-21N, Kobe, Japan).

The dot blot technique was used to detect 3-nitrotyrosine (3-NT), a biomarker for protein tyrosine nitration, in plasma [11,24,25]. EDTA plasma (25 μg per well) was transferred to a Protran BA85 (0.45 μm) nitrocellulose membrane (GE Healthcare, Chicago, IL, USA) via a Minifold I vacuum dot blot system (Whatman Schleicher & Schuell, London, UK). Each slot was washed twice with 200 μL of PBS before and after protein transfer. The membrane was dried for 60 min at 60 °C. Protein tyrosine nitration was detected using a specific antibody for 3-NT (1 μg/mL, Merck Millipore, 06-284, Billerica, MA, USA). Positive dots were visualized by enhanced chemiluminescence after incubation with a peroxidase-coupled secondary antibody (1:10,000, Vector Lab, PI-1000, Burlingame, CA, USA). All incubation and washing steps were performed according to the manufacturer’s instructions. Densitometric quantification of the dots was performed using the PierceTM ECL kit from Thermo Scientific (Waltham, MA, USA) and a ChemiLux Imager (CsX-1400M, Intas, Göttingen, Germany) with Gel-Pro Analyzer software (Media Cybernetics, Bethesda, MD, USA).

Dihydroethidium (DHE)-dependent fluorescence microtopography was used to determine the vascular and cardiac ROS formation in cryosections of the aorta and heart [26,27]. Aortic ring segments (4 mm) and isolated hearts were embedded in aluminum cups containing OCT (optimal cutting temperature)-resin and frozen in liquid nitrogen. The thawed cryosections (8 μm) were incubated with DHE (1 μM in PBS) for 30 min at 37 °C and ROS-derived red fluorescence was detected using a Zeiss Axiovert 40 CFL microscope, Zeiss lenses and Axiocam MRm camera (Oberkochen, Germany).

Oxidative stress from aortic superoxide was complementary measured by a modified HPLC-based method to quantify 2-hydroxyethidium (superoxide-specific oxidation product) levels [28,29]. Aortic tissue was incubated with 50 µM DHE for 30 min at 37 °C in PBS buffer. The tissue was pulverized in a mortar under liquid nitrogen, resuspended in homogenization buffer (acetonitrile/PBS, 1:1), centrifuged and 50 µL of the supernatant was subjected to HPLC analysis. The system consisted of a control unit, two pumps, mixer, detectors, column oven, degasser and an autosampler (AS-2057 plus) from Jasco (Groß-Umstadt, Germany) and a C18-Nucleosil 100-3 (125 × 4) column from Macherey & Nagel (Düren, Germany). A high-pressure gradient was employed with acetonitrile and 50 mM citrate buffer pH 2.2 as mobile phases with the following percentages of the organic solvent: 0 min, 36 %; 7 min, 40 %; 8–12 min, 95 %; 13 min, 36 %. The flow was 1 mL/min and DHE was detected by its absorption at 355 nm whereas the superoxide-specific oxidation product 2-hydroxyethidium and the unspecific oxidation product ethidium was detected by fluorescence (Ex. 480 nm/Em. 580 nm).

### 2.6. Reverse Transcription Polymerase Chain Reaction PCR (qRT-PCR)

Total RNA from aortic tissue was isolated using the acid guanidinium thiocyanate-phenol-chloroform extraction method [30]. Mouse aortas were snap-frozen, transferred to guanidinium thiocyanate (GIT) denaturing solution and tissue was homogenized with the TissueLyser II (Qiagen). A total of 125 ng of total RNA was used for real-time RT-PCR analysis with the QuantiTect Probe RT-PCR kit (Qiagen). 

Probe-and-primer sets purchased from Applied Biosystems were used to analyze the mRNA expression patterns of interleukin 6 (*Il6*, Mm00446190_m1), inducible nitric oxide synthase (*iNos*, Mm00440485_m1), tumor necrosis factor alpha (*Tnfα*, Mm00443259_g1), intercellular adhesion molecule-1 (*Icam1*, Mm00516023_m1) and normalized on the TATA box binding protein (*Tbp*, Mm00446973_m1) as an internal control. Results were quantified with the relative ΔC_t_ method and mRNA target levels were expressed as a percentage of control.

### 2.7. Immunohistochemical Staining of Aortic Rings

Aortic ring segments (3–4 mm) with intact adventitia and perivascular fat were fixed in paraformaldehyde (4%), paraffin-embedded and cut into sections of 5 µm. After deparaffinization, samples were blocked with 2.5 % normal horse serum blocking solution (Vector) and stained with primary antibodies against either NADPH-oxidase 2 (Nox-2, 1:200, LSBio, LS-B12365, Seattle, WA, USA) or 3-NT (1:200, Merck Millipore, 06-284:) and biotinylated with a secondary antibody (1:1000, B-2770, Invitrogen, Carlsbad, CA, USA). The ABC reagent (Vector) with DAB peroxidase substrate (Vector) was used for immunohistochemical detection. The brown precipitate formed by oxidized DAB was visualized using an Olympus IX71 microscope (40× objective) and an Olympus Color View II camera (Shinjuku, Tokyo, Japan). Image J software (NIH, USA) was used to measure the level of expression of Nox2 and 3-NT positive proteins in aortic tissue. For accuracy, measurements were performed twice and averaged by two blinded investigators, values were expressed as the % of stained aortic area.

### 2.8. Statistical Analysis

Results are expressed as mean ± SEM. Statistical calculations were performed with GraphPad Prism 8 (GraphPad Software Inc., San Diego, CA, USA). Two-way ANOVA with Bonferroni’s correction was used for comparisons of concentration–relaxation curves. For prostaglandin F2α-induced maximal constriction, body weight and temperature, blood glucose, mRNA expression, immunohistochemistry, dot blot and oxidative stress and hematology parameters, a one-way ANOVA was used with Tukey’s correction for the comparison of multiple means. In cases of failed normality, the Kruskal–Wallis test (Dunn multiple comparison) was used. *p* values < 0.05 were considered statistically significant and marked by: * vs. B6J and # vs. CLP.

## 3. Results

### 3.1. Treatment with the GLP-1 Receptor Agonist Liraglutide Prevents CLP-Induced Endothelial Dysfunction in Polymicrobial Septic Mice

**Figure 2:**
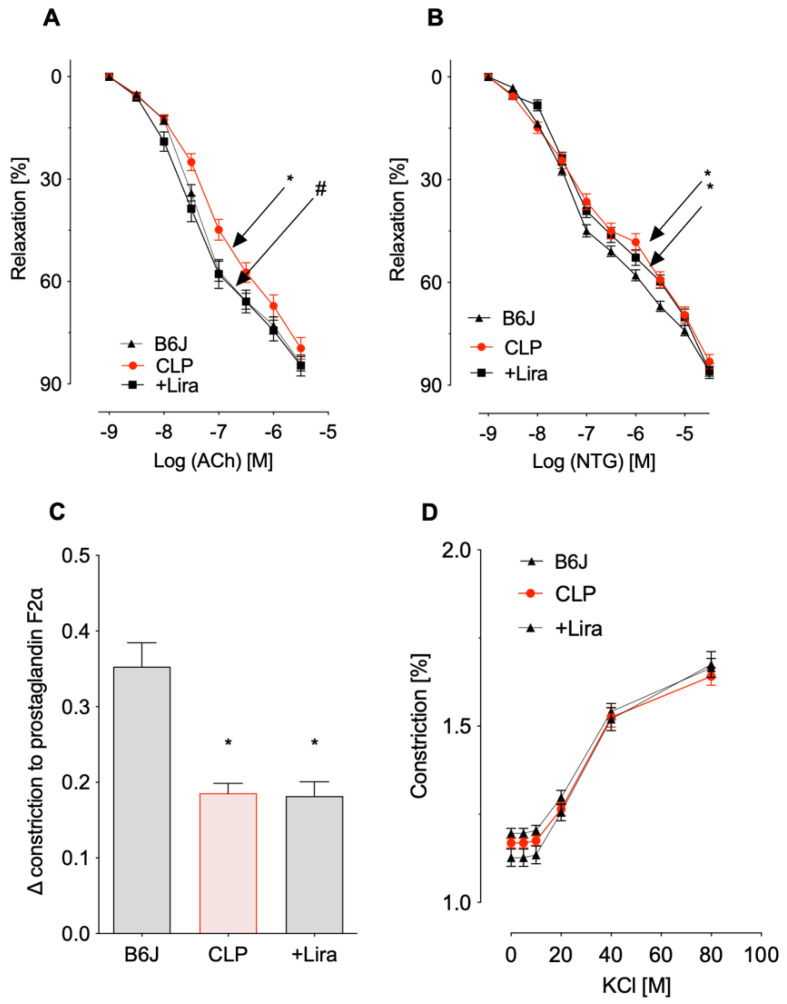
Treatment with the GLP-1 receptor agonist liraglutide prevents CLP-induced endothelial dysfunction in polymicrobial septic mice. (**A**,**B**) Endothelium-dependent (acetylcholine, ACh) and independent (nitroglycerine, NTG) relaxation of thoracic aortic rings was measured in isometric tension recordings. (**C**,**D**) Vasoconstrictor responses prompted by 2 μM prostaglandin F2α and increasing concentrations of KCl. Data are the mean ± SEM of 22–34 (**A**), 21-35 (**B**) or 26–36 (**C**,**D**) independent measurements. * *p* < 0.05 vs. B6J; # *p* < 0.05 vs. CLP.

CLP negatively impacted both endothelium-dependent (ACh-response) and endothelium-independent (NTG-response) vasodilation. Additional treatment of septic mice with liraglutide (+Lira) significantly improved endothelial dysfunction caused by CLP whereas no significant protective effect on NTG-induced smooth muscle relaxation was shown (Figure 2A,B). Maximal constriction prompted by 2 μm prostaglandin F2α (PGF2α) was impaired in aortic rings from both vehicle- and liraglutide-treated CLP mice (Figure 2C). Vasoconstriction in response to depolarizing KCl was not changed by CLP or additional liraglutide administration compared to control mice (Figure 2D).

### 3.2. Treatment with the GLP-1 Receptor Agonist Liraglutide Ameliorates CLP-Induced Complications of Leukopenia and Hyperthermia without Altering Blood Glucose Levels in Polymicrobial Septic Mice

**Figure 3:**
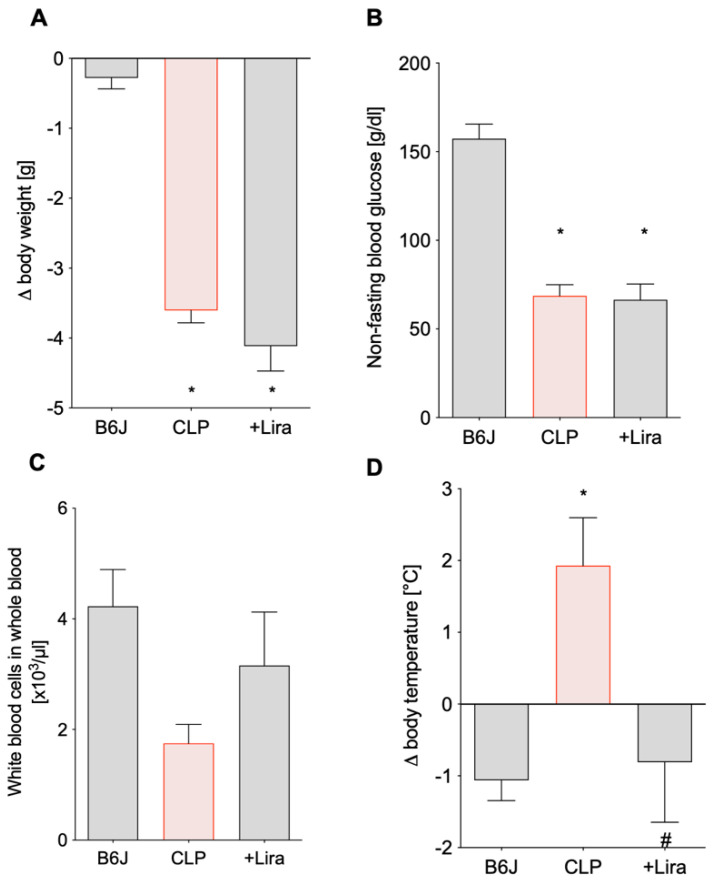
Treatment with the GLP-1 receptor agonist liraglutide protects against CLP-induced complications of leukopenia and hyperthermia without altering blood glucose levels in polymicrobial septic mice. (**A**,**D**) On day 0 and 3, animals were weighed and their temperature taken to determine Δ body weight and Δ body temperature, respectively. (**B**,**C**) Non-fasting blood glucose levels and white blood cell counts were evaluated in whole blood 2 days following sepsis induction. Data are the mean ± SEM of 22–26 (**A**), 19-22 (**B**) and 16-24 (**D**) measurements from individual animals or 6 individual blood samples (each pooled from 3 to 4 mice) (**C**). * *p* < 0.05 vs. B6J; # *p* < 0.05 vs. CLP.

Septic mice significantly lost body weight two days after CLP procedure, which was slightly (but non-significantly) aggravated with additional liraglutide treatment (Figure 3A). Furthermore, both CLP and +Lira group showed significantly reduced non-fasting blood sugar levels (Figure 3B). Mice subjected to vehicle-treated CLP trended towards a lower number of leukocytes (Figure 3C) and showed a drop in platelets (Table 1) in the whole blood while their body temperature significantly increased (Figure 3D). Liraglutide treatment of septic mice was able to normalize the body temperature to control levels (Figure 3D) and white blood cell count and platelet count at least by trend (Figure 3C and Table 1). CLP-induced sepsis led to anemia (decreased red blood cell count and hemoglobin) and microcytosis of erythrocytes (Table 1), all of which was not significantly corrected by liraglutide (although a trend of improvement was observed for some of the parameters).

### 3.3. Treatment with the GLP-1 Receptor Agonist Liraglutide Reduces CLP-Induced Vascular, Cardiac and Systemic Oxidative Stress in Polymicrobial Septic Mice

**Figure 4:**
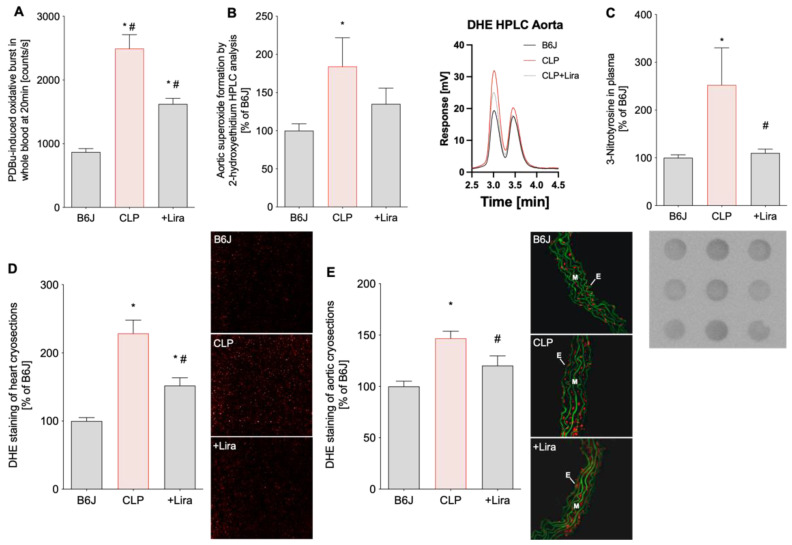
Treatment with the GLP-1 receptor agonist liraglutide reduces CLP-induced vascular, cardiac and systemic oxidative stress in polymicrobial septic mice. (**A**) The whole blood oxidative burst was determined by chemiluminescence (L-012) after phorbol ester (PDBu) stimulation. (**B**) The superoxide-specific DHE product, 2-hydroxyethidium, was quantified in aortic tissue and expressed as a percentage of B6J controls. Representative chromatograms are shown besides the quantification. (**C**) 3-nitrotyrosine (3-NT)-positive proteins in plasma as a consequence of peroxynitrite formation were measured by dot blot analysis. A representative image is shown below the densitometric quantification. (**D**,**E**) Cardiac and aortic cryosections were stained with dihydroethidium to visualize ROS formation as red fluorescence and autofluorescence from aortic laminae as green. The representative photomicrographs are shown next to the densitometric analysis. E, endothelium; M, media. Data are the mean ± SEM of 6 individual blood samples (each pooled from 3 to 4 mice) (**A**), 6-8 (**B**) and 4 (**D**,**E**) measurements from individual animals or 4 individual plasma samples (each pooled from 3 to 4 mice) (**C**). * *p* < 0.05 vs. B6J; # *p* < 0.05 vs. CLP.

Blood oxidative burst and dihydroethidium (DHE)-staining of aortic and cardiac cryosections revealed significantly higher levels of ROS in the whole blood, heart and aorta of septic mice induced by CLP (Figure 4A,D,E). Superoxide-specific oxidation of DHE to 2-hydroxyethidium quantified by HPLC analysis confirmed elevated oxidative stress levels in vehicle-treated CLP-subjected mice (Figure 4B). Additionally, the marker of nitro-oxidative stress, 3-nitrotyrosine (3-NT), was increased in the plasma of CLP-mice (Figure 4C). Importantly, liraglutide treatment was able to significantly mitigate elevated oxidative stress levels of septic mice in all tissues (in the HPLC assay only by trend) and in plasma (Figure 4A–E).

### 3.4. Treatment with the GLP-1 Receptor Agonist Liraglutide Suppresses CLP-Induced Vascular Inflammation in Polymicrobial Septic Mice

**Figure 5:**
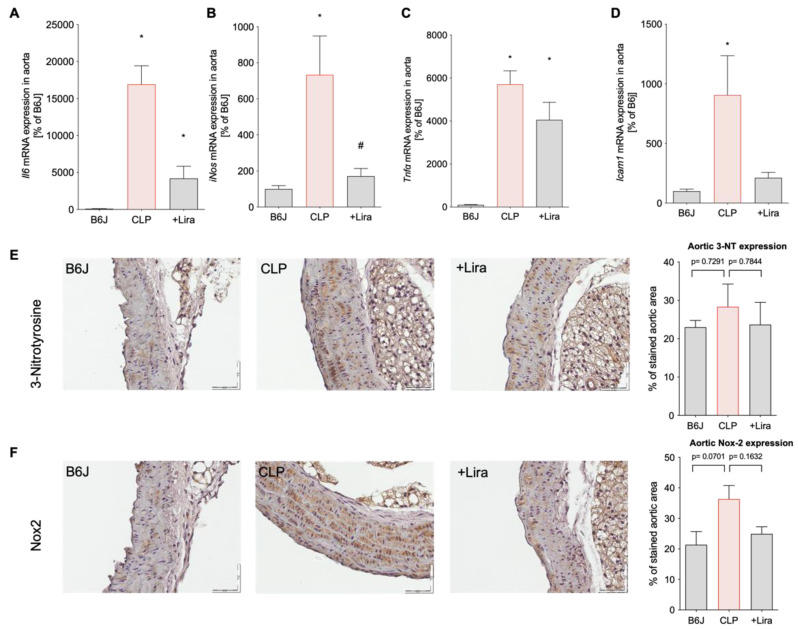
Treatment with the GLP-1 receptor agonist liraglutide suppresses CLP-induced vascular inflammation in polymicrobial septic mice. Aortic gene expression levels of (**A**) interleukin 6 (*Il6*), (**B**) inducible nitric oxide synthase (*iNos*), (**C**) tumor necrosis factor alpha (*Tnfα*) and (**D**) intercellular adhesion molecule-1 (*Icam1*) were measured by quantitative RT-PCR in aortic tissue. (**E**,**F**) Abundance of 3-NT-positive proteins and Nox2 protein expression in the aorta was determined by immunohistochemistry. Representative immunohistochemical images are shown next to the densitometric quantification. Magnification of 40× and scale bar of 50 μm. Data are the mean ± SEM of 3 individual samples (each pooled from 3 to 4 mice) (**A**–**D**) or 3 individual animals (**E**,**F**). * *p* < 0.05 vs. B6J; # *p* < 0.05 vs. CLP.

Cytokine gene expression in the aorta of vehicle-treated septic mice was markedly increased as shown by elevated *Il6* and *Tnfα* mRNA expression levels (Figure 5A,C). Furthermore, intercellular adhesion molecule-1 (*Icam1*) gene expression, indicative of a proinflammatory phenotype of the vascular wall, and inducible nitric oxide synthase (*iNos*) mRNA levels (most likely from infiltrated activated leukocytes) were significantly upregulated in the CLP-group (Figure 5B,D). Immunohistochemical staining revealed high expression of NADPH oxidase 2 (Nox2) and 3-NT positive proteins in the aorta of CLP-mice, denoting infiltration of superoxide-generating myeloid inflammatory cells and peroxynitrite generation as a result of increased iNOS activity and superoxide production, respectively (Figure 5E,F). Liraglutide administration in septic mice markedly reduced the aortic inflammatory burden reflected by lowered *Tnfα*, *Il6*, *iNos* and *Icam1* mRNA levels and attenuated elevated 3-NT and Nox2 protein expression in blood vessels (Figure 5A-E).

## 4. Discussion

**Figure 6:**
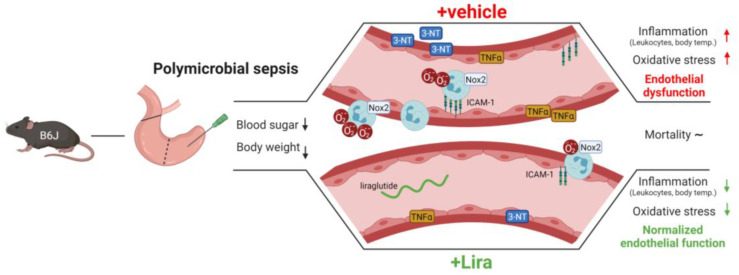
Summary scheme of beneficial cardiovascular effects of the GLP-1 receptor agonist liraglutide on CLP-induced polymicrobial sepsis. CLP-induced mid-grade polymicrobial sepsis led to a reduction in body weight and blood sugar in both vehicle and Lira-treated mice. However, in contrast to the vehicle, Lira protected from sepsis-induced systemic and vascular inflammation and oxidative stress from inflammatory cell-derived Nox2 activity, which translated into a normalized endothelial function yet without a net benefit on mortality. These data demonstrate a new role for GLP-1 receptor agonists like liraglutide in reducing the cardiovascular complications of polymicrobial sepsis based on their antioxidant and anti-inflammatory properties. Created with BioRender.com.

Here we show that the GLP-1 analog liraglutide improved vascular dysfunction, inflammation and oxidative stress in polymicrobial sepsis induced by cecal ligation and puncture (CLP) (Figure 6). CLP led to the loss of body weight and reduced blood glucose, whereas both parameters were not corrected by liraglutide treatment. As a surrogate parameter for a systemic inflammatory response, CLP animals demonstrated elevated body temperature and reduced white blood cell numbers in whole blood, all of which was normalized by liraglutide. Oxidative stress in whole blood and the vascular wall was significantly increased by peritoneal sepsis. Furthermore, induction of proinflammatory genes (*Il6*, *Tnfα*, *Icam1* and *iNos*) and elevated expression the enzyme phagocytic NADPH oxidase (Nox2) and more pronounced oxidative nitration modifications of proteins (3-NT) were observed in septic mice. Importantly, all of these pro-oxidative and proinflammatory phenotypic changes induced by CLP were ameliorated by the application of the GLP-1 analog liraglutide.

GLP-1 is a gut hormone, rapidly degraded by the enzyme dipeptidylpeptidase-4 (DPP-4), with known anti-inflammatory properties. It is released after food uptake from the intestine and is involved in human blood sugar regulation. Analogs of the peptide like liraglutide are established drugs in the treatment of type 2 diabetes mellitus and clinical trials proved a positive effect on the cardiovascular outcome of diabetic patients [31] (for review see [2]). Several preclinical and clinical trials have aimed to elucidate the cardiovascular protective effect of GLP-1 analogs beyond glycemic control. This is complicated by the fact that the GLP-1 receptor is expressed on various cell types including cells of the cardiovascular system of inflammatory cells. Today it is known that not only one single mechanism is relevant for the cardiovascular protection by GLP-1 analogs, several pathways are involved of which the anti-inflammatory action of GLP-1 analogs might play a predominant role [32]. In animal studies it was shown that native GLP-1 and its analogs reduce vascular monocyte adhesion and/or macrophage infiltration within blood vessels [33,34,35]. Proinflammatory cytokines such as TNFα, IL-1β and IL-6 and the inflammatory biomarker C-reactive protein (CRP) have been shown to be downregulated by GLP-1 analogs and inhibitors of the DPP-4 (sitagliptin) [4,36,37,38]. It can be speculated that GLP-1 analogs like liraglutide not only suppresses cytokine release in bacterial septic shock, but also in viral sepsis such as caused by SARS-CoV-2.

Our group investigated the effects of GLP-1 analogs and DPP-4 inhibitors in lipopolysaccharide (LPS)-induced sepsis [11,12]. We demonstrated that DPP-4 inhibitors (linagliptin) and GLP-1 analogs (liraglutide) improved survival in sepsis. Both, DPP-4 inhibition and the GLP-1 analog improved hypotension and endothelial dysfunction in endotoxemic rats. Systemic inflammation was suppressed and vascular oxidative stress was reduced in septic animals treated with liraglutide. Additionally, we observed a positive effect on hemostatic parameters distorted by LPS-induced sepsis (tail bleeding time and activated partial thromboplastin time) [11]. In a subsequent study we used a similar animal model and further investigated effects of GLP-1 analogs on LPS-induced disseminated intravasal coagulation (DIC) and microvascular thrombosis. Thrombocytopenia, microvascular thrombosis in the pulmonary circulation, endothelial dysfunction and increased markers of inflammation in the aorta and whole blood were improved by the GLP-1 analog in LPS-induced sepsis. By using GLP-1R knockout animals we were able to confirm that the beneficial effects of liraglutide on mortality and DIC are mediated via the GLP-1 receptor [12].

The present study is an important addition to the mentioned previously published findings on beneficial effects of GLP-1 analogs in LPS-induced sepsis. Herewith, we used a clinically more relevant sepsis model compared to LPS-induced sepsis, because the latter model has its limitations [15]. In terms of pathophysiology, CLP is comparable to a peritoneal sepsis in which perforation of the intestine leads to the leak of feces into the peritoneum, which results in a fulminant immune response. As we have already demonstrated that the GLP-1 analog liraglutide ameliorates the exaggerated inflammatory reaction in LPS-induced sepsis, we hypothesized that liraglutide has similar effects in CLP-induced sepsis. However, it has to be acknowledged that CLP leads to the effusion of bacteria in the peritoneum with subsequent bacteremia. We thus speculated that suppression of the inflammatory response by liraglutide leads to an adverse effect compared to the findings in LPS-induced sepsis. The present study underlines the potent immunomodulatory action of GLP-1R activation by liraglutide leading to ameliorated vascular dysfunction and reduced oxidative stress/inflammation.

Zaky et al. reported that DPP-4 inhibition by vildagliptin in cholestatic sepsis resulted in increased GLP-1 levels, which reduced fever and ileal NF-κB, TNFα and myeloperoxidase [39]. Similar effects on NF-κB signaling in cardiomyocytes have also been reported for cell culture experiments with LPS stimulation [10]. In angiotensin II-induced arterial hypertension, our own studies revealed that the GLP-1 analog liraglutide has the ability to ameliorate blood pressure and improve endothelial dysfunction by reduction of the infiltration of inflammatory monocytes and neutrophils [40]. This resulted in less oxidative stress and was also associated with a modulation of the NF-κB signaling pathway. We were also able to clarify that this effect is not mediated by a direct effect of GLP-1 on inflammatory cells but on endothelial cells, as the protective effects of liraglutide were lost in endothelial cell specific (*Glp1r^flfl^* × *Cdh5^cre^*) but not in myeloid cell (*Glp1r^flfl^* × *LysM^cre^*) GLP-1R knockout mice [40]. The body temperature lowering effect of liraglutide in CLP-induced sepsis observed in the present study might also be explained by the modulation of NF-κB signaling. Besides the modulation of systemic inflammatory pathways, liraglutide ameliorated vascular inflammation (*Il6*, *iNos*, *Tnfα*, *Icam1* and Nox2) and oxidative stress resulting in improved vascular function (endothelium-dependent relaxation) without impacting the response to vasoconstrictors. The latter findings are similar to previous studies investigating the effects of DPP-4 inhibitors or GLP-1 analogs on improved survival in LPS-induced sepsis by the reduction of systemic and vascular inflammation [11,12]. In this study, aortic Nox2 and *iNos* mRNA expression correlated well with 3-nitrotyrosine (3-NT) expression, suggesting a dominant role for peroxynitrite (ONNO^−^) as a nitrating species. However, we did not investigate a possible contribution of myeloperoxidase in this regard, which has been shown to use nitrite (NO_2_^−^, an autooxidation product of ^•^NO) and hydrogen peroxide (H_2_O_2_) to form reactive nitrogen species also capable of nitrating tyrosine residues in inflamed tissue [41,42,43]. Another limitation is that we did not analyze biological oxidative damage exerted by peroxynitrite other than 3-NT (e.g., lipid peroxidation, sulfoxidation of methionine or cysteine protein residues), which, however, was not a focus of this study.

Interestingly, native GLP-1 is not downregulated in critical illness or myocardial infarction [7,8]. In critically ill patients, GLP-1 levels reflect the severity of the disease and mortality and in acute myocardial infarction it has been shown that GLP-1 levels predict the cardiovascular outcome. It seems as GLP-1 elevation in inflammatory disease such as critical illness or acute myocardial infarction is not causal for the increased mortality or poor cardiovascular outcome, but might be a counteracting mechanism in proinflammatory situations via upregulation of the GLP-1/GLP-1R pathway [44]. Application of GLP-1 analogs with higher plasma levels and longer half-life activates the above-mentioned anti-inflammatory pathways and ameliorates adverse effects of an exaggerated inflammatory response in CLP-induced sepsis. 

This study has some limitations. In LPS-induced sepsis, we performed survival studies and demonstrated that pharmacological DPP-4 inhibition or genetic deletion and administration of the GLP-1 analog liraglutide improved mortality [11]. Due to ethical considerations we decided to not perform survival studies in CLP-induced sepsis as this would have required at least another 40–50 CLP mice with and without the liraglutide treatment. However, based on the included mice in this study, we did not detect any difference in survival in CLP-induced sepsis by treatment with liraglutide (3 of a total of 30 mice died in the CLP group and 5 of a total of 27 mice died in the +Lira group). It can be assumed that without an antibiotic cotreatment there is no survival benefit in CLP-induced sepsis (in contrast to LPS-induced sepsis) since the bacteremia is not treated. However, this study was not designed to answer this hypothesis and further research would be needed. Methodologically, L-012 has been discredited for superoxide formation as a byproduct of redox-cycling in the presence of peroxidases [45]. However, in our hands, it seems suitable for the phorbol ester or endotoxin (e.g., PDBu, zymosan A)-stimulated oxidative burst of isolated leukocytes or whole blood, which largely results from NADPH oxidase derived superoxide and subsequent hydrogen peroxide formation as the L-012 ECL signal is absent in the whole blood of gp91phox deficient mice [22,46]. A general limitation of the study is a reduced sample size in some experiments of only three to four animals, the cytokine measurements on mRNA and not protein level and the study of male mice only to exclude the known effects of sexual hormones on vascular function [47]. Despite these limitations, liraglutide is an approved drug with few known side effects and low treatment costs. For clinical use, a placebo controlled double-blind randomized trial would be needed to test the beneficial effect of GLP-1 analogs in septic patients.

## 5. Conclusions

In the present study we can confirm that the GLP-1 analog liraglutide protects the cardiovascular system not only in LPS-induced endotoxemia, but also polymicrobial sepsis induced by CLP (Figure 6). Sepsis impairs the vascular function by increased vascular inflammation and oxidative stress. The GLP-1 analog liraglutide displays both antioxidant and anti-inflammatory properties conferring vasoprotection in polymicrobial septic mice. Besides antimicrobial treatment in sepsis, the adjuvant therapy of exaggerated inflammation may represent a future therapeutic strategy, despite that many anti-inflammatory therapeutic approaches failed in the past. Our present results warrant further research on the use of GLP-1 analogs as an adjuvant therapy in septic patients.

## Figures and Tables

**Table 1:** Hematological parameters. Data are the mean ± SEM of 5–7 individual blood samples (each pooled from 3 to 4 mice). * *p* < 0.05 vs. B6J.

Parameter	B6J	CLP	+Lira
WBC (×10^3^/µL)	4.2 ± 0.7	1.8 ± 0.3	3.2 ± 1.0
RBC (×10^6^/µL)	6.87 ± 0.18	5.05 * ± 0.33	5.76 ± 0.52
HGB (g/dL)	10.7 ± 0.2	8.1 * ± 0.5	9.0 ± 0.7
HCT (%)	32.9 ± 0.8	23.4 * ± 1.7	26.8 ± 2.5
MCV (fL)	47.9 ± 0.2	46.1 * ± 0.5	46.5 * ± 0.3
MCH (pg)	15.6 ± 0.1	16.0 ± 0.3	15.6 ± 0.2
MCHC (g/dL)	32.5 ± 0.2	34.7 * ± 0.8	33.6 ± 0.5
PLT (×10^3^/µL)	845 ± 84	381 * ± 53	451 * ± 81
Lym% (%)	73.4 ± 4.2	61.8 ± 10.4	44.5 * ± 8.2
Lym (×10^3^/µL)	4.0 ± 1.0	1.0 ± 0.2	1.7 ± 0.8
RDW_SD (fL)	27.2 ± 0.3	27.2 ± 0.2	27.0 ± 0.2
RDW_CV (%)	12.7 ± 0.4	12.8 ± 0.2	12.3 ± 0.3
PDW (fL)	6.6 ± 0.3	7.2 ± 0.3	7.1 ± 0.3
MPV (fL)	5.7 ± 0.2		6.2 ± 0.2
P_LCR (%)	2.4 ± 0.4	4.5 ± 0.8	4.9 ± 0.9

Abbreviations: WBC, white blood cells; RBC, red blood cells; HBG, hemoglobin; HCT, hematocrit; MCV, mean corpuscular volume; MCH, mean corpuscular hemoglobin; MCHC, mean corpuscular hemoglobin concentration; PLT, platelet count; Lym%, percentage of lymphocytes; Lym, lymphocytes; RDW_SD, red cell distribution width—standard deviation, RDW_CV, red cell distribution width—coefficient of variation; PDW, platelet volume distribution width; MPV, mean platelet volume; P_LCR, platelet larger cell ratio.

## Data Availability

The data presented in this study are all contained within this article.

## Abbreviations

Non-standard Abbreviations and Acronyms	
3-NT	3-nitrotyrosine
ACh	acetylcholine
CLP	cecal ligation and puncture
DHE	dihydroethidium
DPP-4	dipeptidylpeptidase-4
GLP-1	glucagon-like peptide-1
GLP-1R	glucagon-like peptide-1 receptor
IL-6	interleukin 6
iNOS	inducible ^•^NO synthase (type 2)
L-012	8-amino-5-chloro-7-phenylpyrido[3,4-d]pyridazine-1,4-(2H,3H)dione sodium salt
Lira	liraglutide
LPS	lipopolysaccharide
NF-κB	nuclear factor NF-kappa-B
NTG	nitroglycerine
Nox	NADPH oxidase
PDBu	phorbol dibutyrate
ROS	reactive oxygen species
TNFα	tumor necrosis factor alpha
ICAM-1	intercellular adhesion molecule-1
